# Comprehensive analysis and validation of SNX7 as a novel biomarker for the diagnosis, prognosis, and prediction of chemotherapy and immunotherapy response in hepatocellular carcinoma

**DOI:** 10.1186/s12885-023-11405-0

**Published:** 2023-09-25

**Authors:** Jianlin Chen, Gan Gao, Yi Zhang, Peng Dai, Yi Huang

**Affiliations:** 1 Shengli Clinical Medical College, Fujian Medical University, Fuzhou, Fujian, 350001 China; 2 Department of Clinical Laboratory, Fujian Provincial Hospital, Fuzhou, Fujian, 350001 China; 3 Central Laboratory, Fujian Provincial Hospital, Fuzhou, Fujian, 350001 China; 4 Center for Experimental Research in Clinical Medicine, Fujian Provincial Hospital, Fuzhou, Fujian, 350001 China; 5 Departments of Clinical Laboratory of Liuzhou Hospital of Guangzhou Women and Children’s Medical Center, Liuzhou, 545616 Guangxi China; 6 Departments of Anesthesiology, The First People’s Hospital of Foshan, Foshan, 528000 Guangdong China

**Keywords:** Sorting nexin 7, Biomarker, Hepatocellular carcinoma, Diagnostic, Prognosis

## Abstract

**Background:**

Studies have demonstrated that Sorting nexin 7 (SNX7) functions as an anti-apoptotic protein in liver tissue and plays a crucial role in the survival of hepatocytes during early embryonic development. However, its diagnostic and prognostic value as well as the predictive value of chemotherapy and immunotherapy have not been reported in hepatocellular carcinoma (HCC).

**Methods:**

SNX7 mRNA expression and its diagnostic efficacy were examined in GEO datasets, and the findings were further confirmed in TCGA, ICGC cohorts, and cell lines. The protein level of SNX7 was determined using CPTAC and HPA databases, and the results were validated through immunohistochemistry (IHC). Survival analyses were performed in TCGA and ICGC cohorts, and the results were subsequently validated via Kaplan–Meier Plotter. The response to chemotherapy and immunotherapy was predicted via GDSC dataset and TIDE algorithm, respectively. R packages were employed to explore the relationship between SNX7 expression and immune infiltration, m6A modification, as well as the functional enrichment of differentially expressed genes (DEGs).

**Results:**

The expression of SNX7 at both mRNA and protein levels was significantly upregulated in HCC tissues. SNX7 exhibited superior diagnostic efficacy compared to AFP alone for HCC detection, and combining it with AFP improved the diagnostic accuracy for HCC. High SNX7 was associated with unfavorable outcomes, including poor overall survival, disease-specific survival, progression-free survival, and advanced pathological stage, in patients with HCC, and SNX7 was identified as an independent risk factor for HCC. Moreover, elevated SNX7 expression was positively correlated with increased sensitivity to various chemotherapy drugs, including sorafenib, while it was associated with resistance to immunotherapy in HCC patients. Correlation analysis revealed a relationship between SNX7 and multiple m6A-related genes and various immune cells. Finally, enrichment analysis demonstrated strong associations of SNX7 with critical biological processes, such as cell cycle regulation, cellular senescence, cell adhesion, DNA replication, and mismatch repair pathway in HCC.

**Conclusions:**

Our study highlights the association of SNX7 with the immune microenvironment and its potential influence on HCC progression. SNX7 emerges as a promising novel biomarker for the diagnosis, prognosis, and prediction of response to chemotherapy and immunotherapy in patients with HCC.

**Supplementary Information:**

The online version contains supplementary material available at 10.1186/s12885-023-11405-0.

## Introduction

Hepatocellular carcinoma (HCC) accounts for more than 90% of primary liver cancer cases and represents the main histological type of liver cancer [1]. It constitutes approximately 6% of all human cancers [2] and ranks second globally in terms of cancer-related mortality, posing a major challenge to public health. Despite significant advancements in research and clinical efforts, the prognosis for patients with advanced HCC remains unsatisfactory. Therefore, it is critically needed to identify new and reliable predictors for the clinical treatment and improvement of prognosis of patients with liver cancer.

Sorting nexin 7 (SNX7), an early endosome and multivesicular-body-distributed protein, is one of the members of the sorting nexin (SNX) family that plays vital roles in various intracellular biological processes, such as endocytosis, protein sorting, and endosomal signaling [3–5]. Previous research has demonstrated the importance of SNX7 in hepatocyte survival during early embryonic formation in zebrafish, where it functions as an anti-apoptotic protein abundant in liver tissue [6]. SNX7 has also been implicated in Alzheimer’s disease (AD), with genetic associations found and decreased expression observed in AD subjects [7, 8]. Additionally, overexpression of SNX7 has been shown to reduce the production of amyloid-βpeptides (Aβ) by enhancing lysosomal degradation of amyloid precursor protein (APP) in HEK293T. Recent studies have inferred that aberrant SNX7 expression may have potential clinical implications in predicting lung cancer. For instance, one study developed a predictive model for recurrence in early-stage lung adenocarcinoma, incorporating SNX7 alongside other epithelial-to-mesenchymal transition-related genes (AGL, ECM1, ENPP1, SNX7, and TSPAN12) [9]. Another study identified SNX7 as an unfavorable prognosis gene in lung adenocarcinoma [10]. Nevertheless, the clinical value of its diagnosis, prognosis, and prediction of susceptibility to drug therapy in HCC has not been described.

The present research systematically investigated the expression profile of SNX7 in HCC and its association with pathological features and clinical outcomes using multiple datasets. Firstly, we analyzed expression profiles and potential diagnostic values of SNX7 in various GEO datasets, which was proved in external TCGA and ICGC cohorts. Moreover, the protein level of SNX7 was determined using data from CPTAC, and further validated by HPA. Subsequently, we evaluated its potential prognostic value in TCGA and ICGC cohorts and confirmed by Kaplan–Meier plotter. Additionally, the study assessed the predictive value of SNX7 expression for HCC patient response to chemotherapy and immunotherapy. In addition, the correlation between SNX7 expression and immune infiltration, immune checkpoint genes, and m6A-related genes were subsequently investigated. Finally, enrichment analysis was conducted. These findings provide important insights into the potential significance of SNX7 in HCC.

## Materials and methods

### Data collection and analysis

**Fig. 1:**
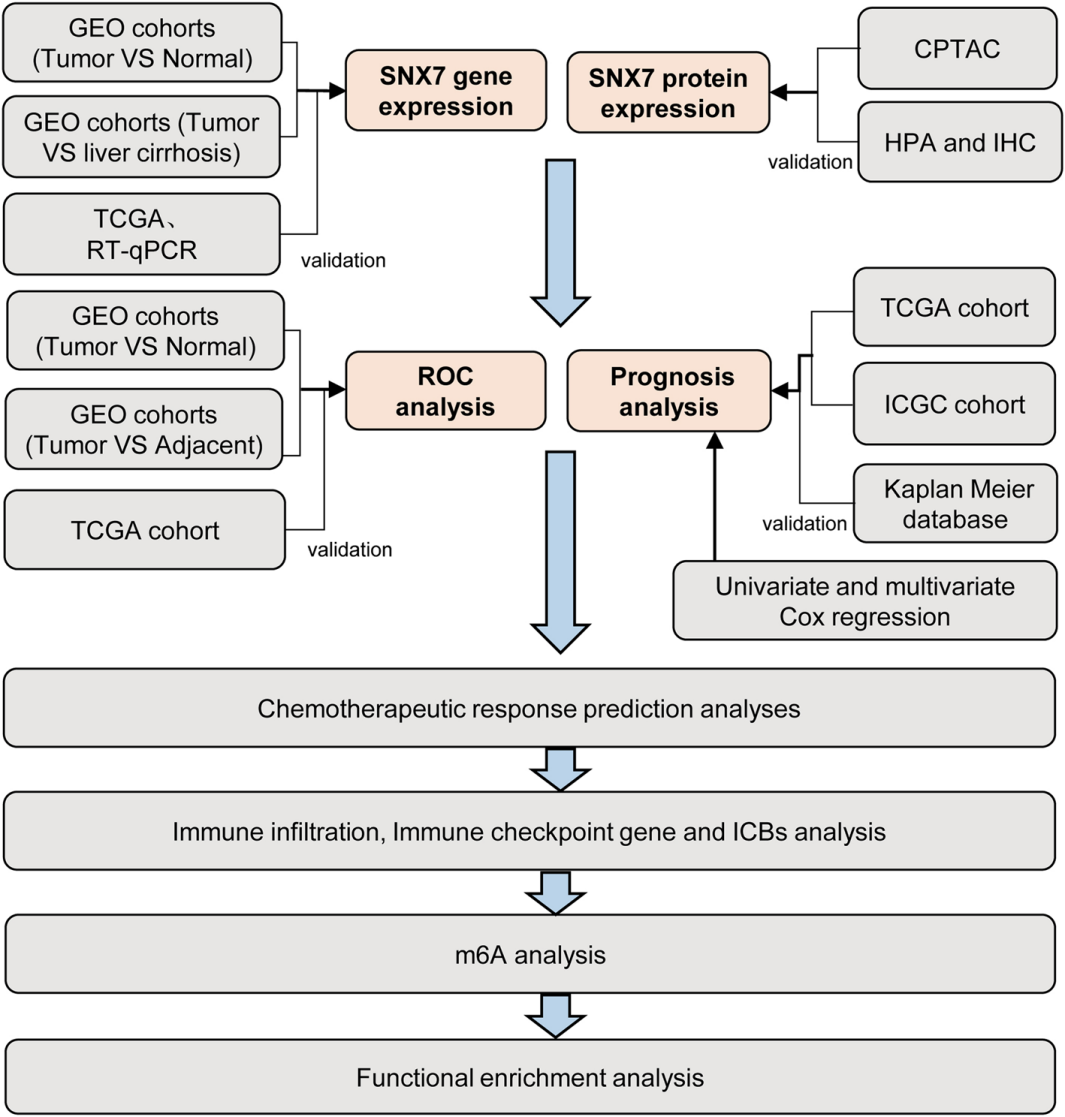
Flow chart of the study

The RNA-sequencing data from multiple GEO datasets (GSE144269 [11], GSE45267 [12], GSE112790 [13] and GSE14520 [14]) were obtained from the Gene Expression Omnibus (GEO) database(www.ncbi.nlm.nih.gov/geo).Then, differences in SNX7 transcriptome levels were analyzed between HCC and normal liver tissues. Similarly, based on five datasets (GSE121248 [15], GSE10143 [16], GSE36376 [17], GSE76427 [18], and GSE39791 [19]), the mRNA level differences of SNX7 between HCC and adjacent normal liver tissue were examined subsequently. Additionally, three datasets (GSE25097 [20], GSE46444 [21], and GSE54236 [22]) were also downloaded for the analysis of the difference of SNX7 gene expression between HCC and liver cirrhosis tissues. Moreover, HCC and normal samples with normalized expression data as well as relevant clinical features were downloaded from TCGA (http://cancergenome.nih.gov) and GTEX databases (https://www.gtexportal.org/home/). We also downloaded the RNA-sequencing data and relevant clinical data from the International Cancer Genome Consortium (ICGC) (LIRI-JP) cohort [23]. The data analyses were conducted in accordance with previous publications [24–28]. A flow diagram of current research can be seen in Fig. 1.

The protein expression of SNX7 was assessed using the publicly available online tool UALCAN [29] through the CPTAC data portal (https://cptac-data-portal.georgetown.edu/cptacPublic/) and validated by the HPA database (https://www.proteinatlas.org/).

### Cell culture and RT–qPCR

Human normal liver cell line (LO2) and human liver cancer cell lines (HuH-1, HuH-7 and HepG2) were purchased from Hongshun Biotechnology Co. LTD (Shanghai, China). LO2 cells were cultured in DMEM (Gibco, CA, USA) supplemented with 20%FBS (Procell, Wuhan, China). HuH-1, HuH-7 and HepG2 cells were cultured in RPMI-1640 (Gibco, CA, USA) supplemented with 10%FBS. All cells were maintained in a 5% CO2 incubator humidified at 37℃.

Total RNA was isolated from the cells using the SteadyPure Quick RNA Extraction Kit (Accurate Biology, AG21023, China) according to the manufacturer’s manual. cDNA was synthesized by GoScript™ Reverse Transcription System (A5001, Promega). qPCR was performed using the GoTaq® Master Mix (A6001, Promega) was used for qPCR. Primers for SNX7 were synthesized by Shangya Biotechnology (Fuzhou, China) with the following sequences: forward: 5ʹ-GCCCTGAAAGCAGATTGGGAG-3ʹ, reverse: 5ʹ- AGGCTTCTTCCAAGTGAAGGT-3ʹ. The 2^−ΔΔCT^ method was used to calculate the relative expression levels of SNX7 with GAPDH as a reference gene.

### Tumor tissues specimens and immunohistochemistry

Tissue specimens were fixed in 10% formalin and embedded in paraffin. The tissues were then cut into 3um thick sections. After dewaxing and hydration, citric acid buffer (0.01M, pH 6.0) was used and boiled for 15 min for antigen repair. Immunohistochemically staining was conducted using the EliVision™Plus kit (Maixin Biotechnology, Fuzhou, China). Subsequently, the sections were incubated overnight at 4℃ with an anti-SNX7 polyclonal antibody (12269–1-AP) (1:200) or with PBS as a negative control. After incubation, the sections were treated with secondary antibody at room temperature for 10 min and then stained with diaminobenzidine (DAB Kit, Lab Vision) for 40 s. Hematoxylin was used to counterstain the cells showing a positive immunohistochemical reaction for 15 s. Finally, the sections were dehydrated and dried and examined under a microscope.

### Diagnostic and prognostic value of SNX7 in LIHC

The area under the curve (AUC) values for the receiver operating curve (ROC) were calculated and plotted using the “pROC” package and “ggplot2” package in R version 3.6.3. Survival data, made available from ICGC Data and TCGA portals, were analyzed with survival and “survminer” R packages [30]⁠, and validated by the Kaplan Meier plotter [31] with the median of SNX7 expression as the optimal cutoff point.

### Chemotherapeutic response prediction

As previously described [32, 33], the response to chemotherapy was evaluated using the Genomics of Drug Sensitivity in Cancer (GDSC) data. The half maximal inhibitory concentration (IC50) value was used to reflect the drug sensitivity, and the lower the value, the more sensitive to the drug.

### Analysis of immune cell infiltration, immune checkpoint gene and (immune checkpoint blockade, ICB)

According to previous reports, immune infiltration of 24 types of immune cells was analyzed using ssGSEA implementation in the GSVA R package [34, 35]. The enrichment score was calculated using ssGSEA, and its value was adopted to represent the level of immune infiltration. After that, the correlation between SNX7 expression level and immune cell infiltration was analyzed by Spearman.

The relationships between SNX7 and common immune checkpoint-related genes were carried out according to a previous report [36]. Potential ICB response was predicted with TIDE algorithm [37].

### Analysis of m6A related gene

Twenty m6A-related genes were derived from a previously published literature [38]. The result is then implemented by the R Foundation (version 4.0.3) packages GGplot2 and pheatmap.

### Functional enrichment analyses

Enrichment analysis was implemented via the “clusterProfiler” package to explore the potential functional annotations and pathways based on the differentially expressed genes (DEGs) [39]. *p* value < 0.05 or FDR < 0.05 was regarded as meaningful.

### Statistical analysis

R language software (version 4.0.3 & 3.6.3) from the official website (https://www.r-project.org/) was utilized for the analysis in this study. To assess the differences between two groups, the Wilcoxon rank sum test or paired t-test was employed. For comparing differences among multiple groups, Kruskal–Wallis rank-sum test and Dunn’s test was used. The overall survival disparities between groups were evaluated through Kaplan–Meier analysis and a log-rank test. The diagnostic value of SNX7 or AFP for HCC was evaluated with receiver operating characteristic curve (ROC). Spearman’s correlation test was performed to determine the association between the expression of SNX7 and levels of immune cell infiltration. Results were considered statistically significant when the *p*-value was less than 0.05.

## Result

### SNX7 is significantly upregulated in HCC and associated with progression of HCC

**Fig. 2:**
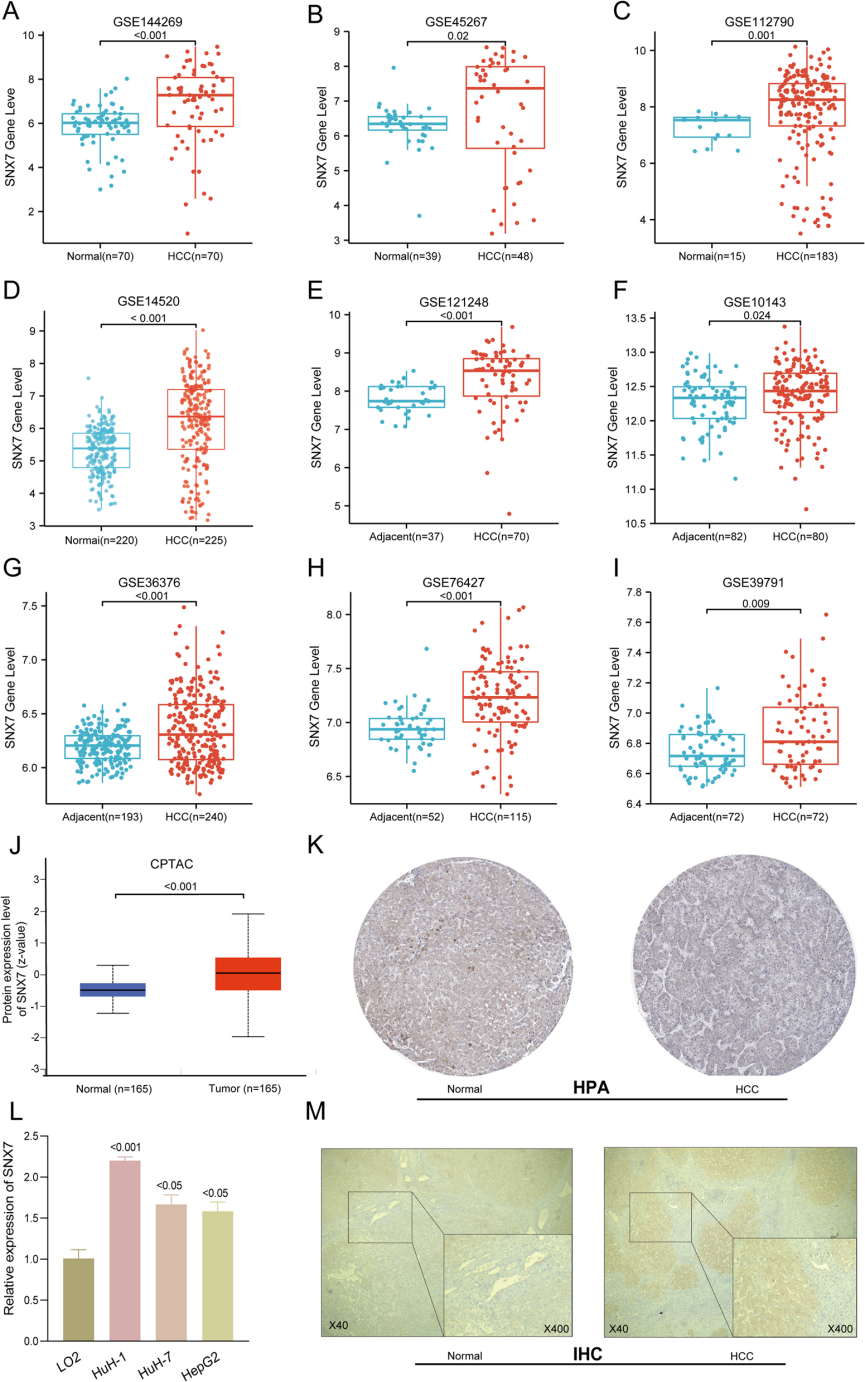
SNX7 expression profiles in hepatocellular carcinoma. The SNX7 transcript levels in tissues of HCC and normal samples based on (**A**) GSE144269; **B** GSE45267; **C** GSE112790; **D** GSE14520. The SNX7 mRNA levels in HCC and adjacent normal liver tissues based on (**E**) GSE121248; **F** GSE10143; **G** GSE36376; **H** GSE76427; **I** GSE39791. **J** The protein expression of SNX7 in HCC and normal liver specimens from CPTAC data. **K** Validation of protein expression of SNX7 based on the HPA database. **L** Validation of gene expression of SNX7 between human normal liver cell line (LO2) and HCC cell lines. **M** Validation of protein expression of SNX7 based on IHC. Analysis between two groups of samples: Wilcoxon rank sum test

**Fig. 3:**
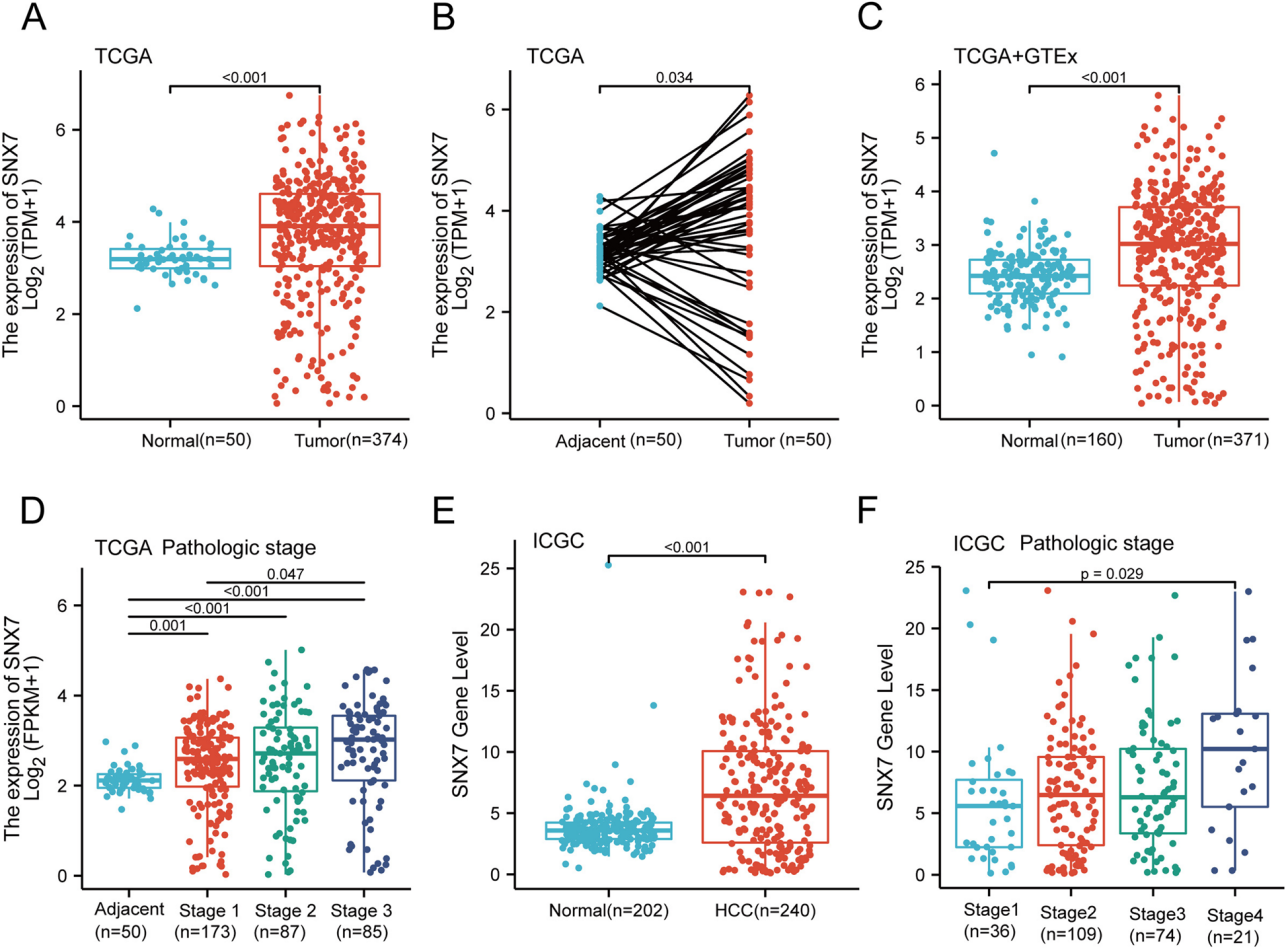
SNX7 mRNA expression correlates with HCC pathologic stages. **A**-**C** High mRNA levels of SNX7 in HCC validated in the analysis of data from TCGA and GTEx database. **D** Box plot showing SNX7 mRNA expression in 1–3 pathologic stages of HCC specimens from TCGA datasets. **E** Validation of the mRNA levels of SNX7 in the ICGC cohort. **F** Validation of the SNX7 mRNA expression in 1–4 pathologic stages of HCC specimens from ICGC cohort. Analysis between two groups of samples: Wilcoxon rank sum test. Significances between multiple groups of samples were determined by Kruskal–Wallis rank-sum test and Dunn’s test

Our analysis of gene expression using RNA sequencing data obtained from GEO revealed significantly higher mRNA levels of SNX7 in HCC compared to normal tissues in multiple datasets, including GSE144269, GSE45267, GSE112790 and GSE14520 (Fig. 2A-D, all *p* < 0.05). Similarly, SNX7 levels were found to be increased in HCC compared to adjacent normal liver tissues in GSE121248, GSE10143, GSE36376, GSE76427 and GSE39791 datasets (Fig. 2E-I, all *p* < 0.05). These findings were further validated in TCGA and GTEx datasets as well as ICGC (LIRI-JP) cohort (Fig. 3A-C, Fig. 3E, all *p* < 0.05). In addition, significant differences in SNX7 protein expression were observed between normal and HCC tissues from CPTAC data (Fig. 2J, *p* < 0.05), which was further validated by HPA (Fig. 2K) and by IHC (Fig. 2M). RT-qPCR results also confirmed higher SNX7 expression in HCC cell lines, consistent with the bioinformatics analysis (Fig. 2L).

**Fig. 4:**
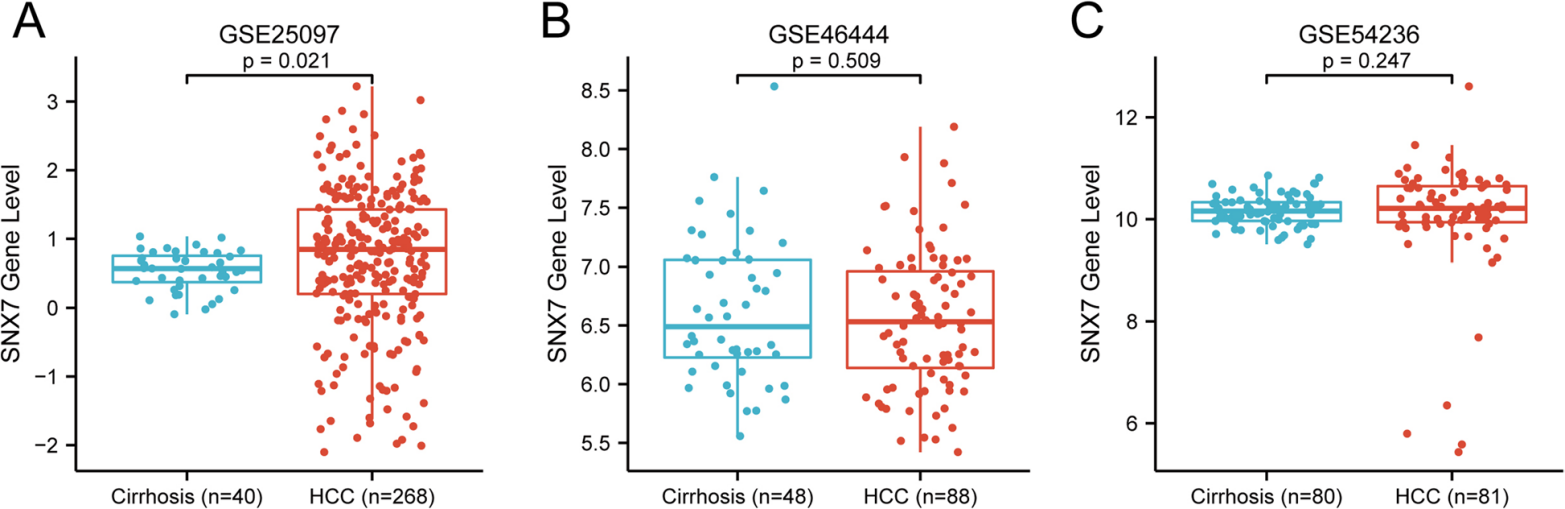
The mRNA expression level of SNX7 between HCC patients and liver cirrhosis patients. The analysis of SNX7 levels in (**A**) GSE25097, (**B**) GSE46444, and (**C**) GSE54236 grouped by HCC and liver cirrhosis tissues. The difference between two groups was analyzed using the Wilcoxon rank sum test

Based on the TCGA database, we found that SNX7 expression were significantly higher in stage 3 liver cancer compared to stage 2 and stage 1(Fig. 3D, all *p* < 0.05). Similarly, the level of SNX7 was significantly higher in stage 4 of HCC than that in stage 1 HCC samples from the ICGC cohort (Fig. 3F, *p* < 0.05). Furthermore, we found significantly higher SNX7 mRNA levels in HCC compared to liver cirrhosis samples (Fig. 4A, *p* < 0.001). However, this difference was not observed in GSE46444 and GSE54236 datasets (Fig. 4B-C, all *p* > 0.05). The above findings indicated that SNX7 is overexpressed in HCC and positively correlated with the development of hepatocellular carcinoma.

### SNX7 is a novel diagnosis and prognosis biomarker for HCC

**Fig. 5:**
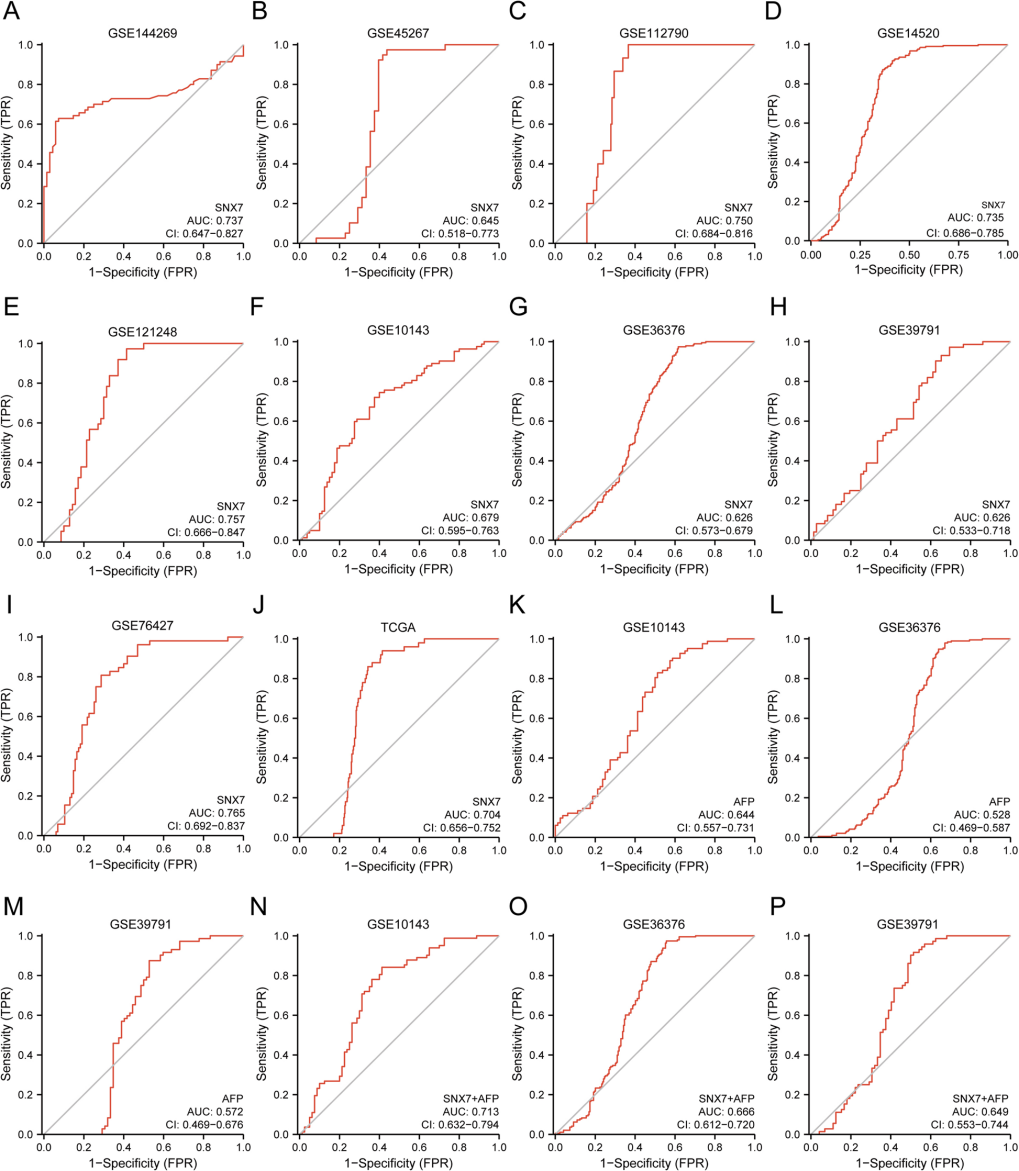
ROC curve of SNX7 mRNA expression in different LIHC cohorts. The ROC curves of SNX7 in distinguish and normal in GSE144269 (**A**), GSE45267 (**B**), GSE112790 (**C**), and (**D**) GSE14520. ROC curves of SNX7 for differentiating HCC tissues and adjacent normal tissues in GSE121248 (**E**), GSE10143 (**F**), GSE36376 (**G**), GSE39791 (**H**), and GSE76427 (**I**). ROC curves of SNX7 in validation sets of TCGA datasets (**J**). ROC curves of AFP for differentiating HCC tissues and adjacent normal tissues in GSE10143 (**K**), GSE36376 (**L**), and GSE39791 (**M**). ROC curves of SNX7&AFP for differentiating HCC tissues and adjacent normal tissues in GSE10143 (**N**), GSE36376 (**O**), and GSE39791 (**P**)

Next, we assessed and validated the diagnostic value of SNX7 in multiple datasets. First of all, we analyzed the capacity of SNX7 in the separation of liver cancer from normal samples based on the GSE144269, GSE45267, GSE112790 and GSE14520 datasets. The results showed that the AUCs of SNX7 were 0.737, 0.645, 0.750, and 0.735, respectively (Fig. 5A-D). ROC curves for SNX7 expression in distinguishing HCC tissue from adjacent normal tissues were shown in Fig. 5E-I, with AUC values of 0.757, 0.679, 0.626, 0.626, and 0.765, respectively. Then, diagnostic ROC curves of SNX7 for distinguishing HCC from normal samples were validated in TCGA (TCGA tumor vs TCGA normal) with AUC value of 0.704 (Fig. 5J). These results strongly supported that SNX7 was a potential diagnostic biomarker for HCC. Moreover, the AUC values of 0.679, 0.626 and 0.626 for SNX7 were respectively significantly higher than the AUC values of 0.644, 0.528 and 0.572 for AFP distinguishing HCC from adjacent normal tissues (Fig. 5K-M). It was conclusion that SNX7 was a more powerful diagnostic marker than AFP for HCC. In addition, when SNX7 and AFP were combined for HCC detection, the value of AUCs go up to 0.713, 0.666, and 0.649, respectively (Fig. 5N-P).

**Fig. 6:**
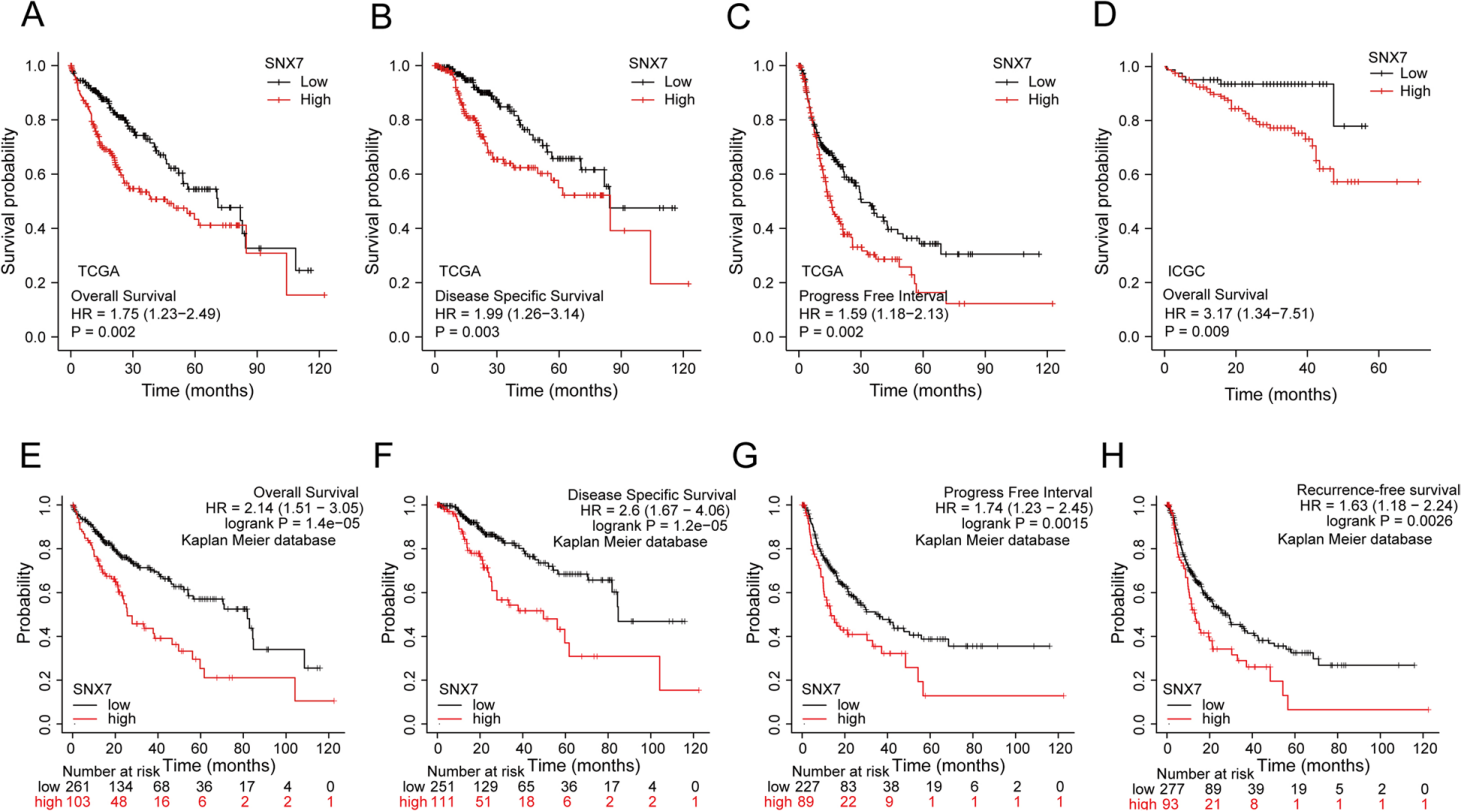
High SNX7 expression predicts poor prognosis in HCC patients. The OS (**A**), DSS (**B**), and PFS (**C**) of HCC patients were analyzed in TCGA. Kaplan–Meier curves for the patients in high- and low-SNX7 groups from ICGC database (**D**). Kaplan–Meier analysis of OS (**E**), DSS (**F**), PFS (**G**), and RFS (**H**) in the validation cohort of the Kaplan Meier database. All survival curves, groups were defined using median split of SNX7 expression

To evaluate the prognostic significance of SNX7 for patients with HCC, KM curves were plotted via TCGA and ICGC datasets, and validated in Kaplan Meier database. The result indicated that high SNX7 mRNA expression was associated with poor OS (*p* = 0.002; HR = 1.75), DSS (*p* = 0.003; HR = 1.99), and PFI (*p* = 0.002; HR = 1.59) in TGCA-LIHC dataset (Fig. 6A-C). Overall survival analysis in ICGC cohort revealed a result like that of the TGCA dataset (*p* = 0.009; HR = 3.17) (Fig. 6D). Results from validation of Kaplan Meier database indicated that HCC patients in SNX7^high^ group were associated with worse OS (log rank *p* = 1.4e-05; HR = 2.14), DSS (log rank *p* = 1.2e-05; HR = 2.6), PFI (log rank *p* = 0.0015; HR = 1.74) and RFS (log rank *p* = 0.0026; HR = 1.63) compared to those in the SNX7^low^ group (Figs. 6E-H). Furthermore, the univariate analysis in TCGA showed that SNX7 was an independent risk factor for LIHC (Table 1). From these results, we can see that the abnormal expression of SNX7 may be closely related to the prognosis of LIHC patients.

**Table 1:** Cox regression analysis for clinical outcomes in LIHC patients

Characteristics	Univariate analysis		Multivariate analysis	
	**Hazard ratio (95% CI)**	***p* value**	**Hazard ratio (95% CI)**	***p* value**
SNX7	1.753 (1.234–2.491)	**0.002**	1.487 (1.003–2.205)	**0.049**
Tumor status	2.317 (1.590–3.376)	**< 0.001**	1.730 (1.153–2.596)	**0.008**
T stage	2.598 (1.826–3.697)	**< 0.001**	1.708 (0.233–12.546)	0.599
Pathologic stage	2.504 (1.727–3.631)	**< 0.001**	1.237 (0.169–9.035)	0.834
Age	1.205 (0.850–1.708)	0.295		
Gender	0.793 (0.557–1.130)	0.200		
Vascular invasion	1.344 (0.887–2.035)	0.163		

*p*-values below 0.05 are bolded

### HCC patients with High SNX7 expression are more sensitive to chemotherapy drugs

**Fig. 7:**
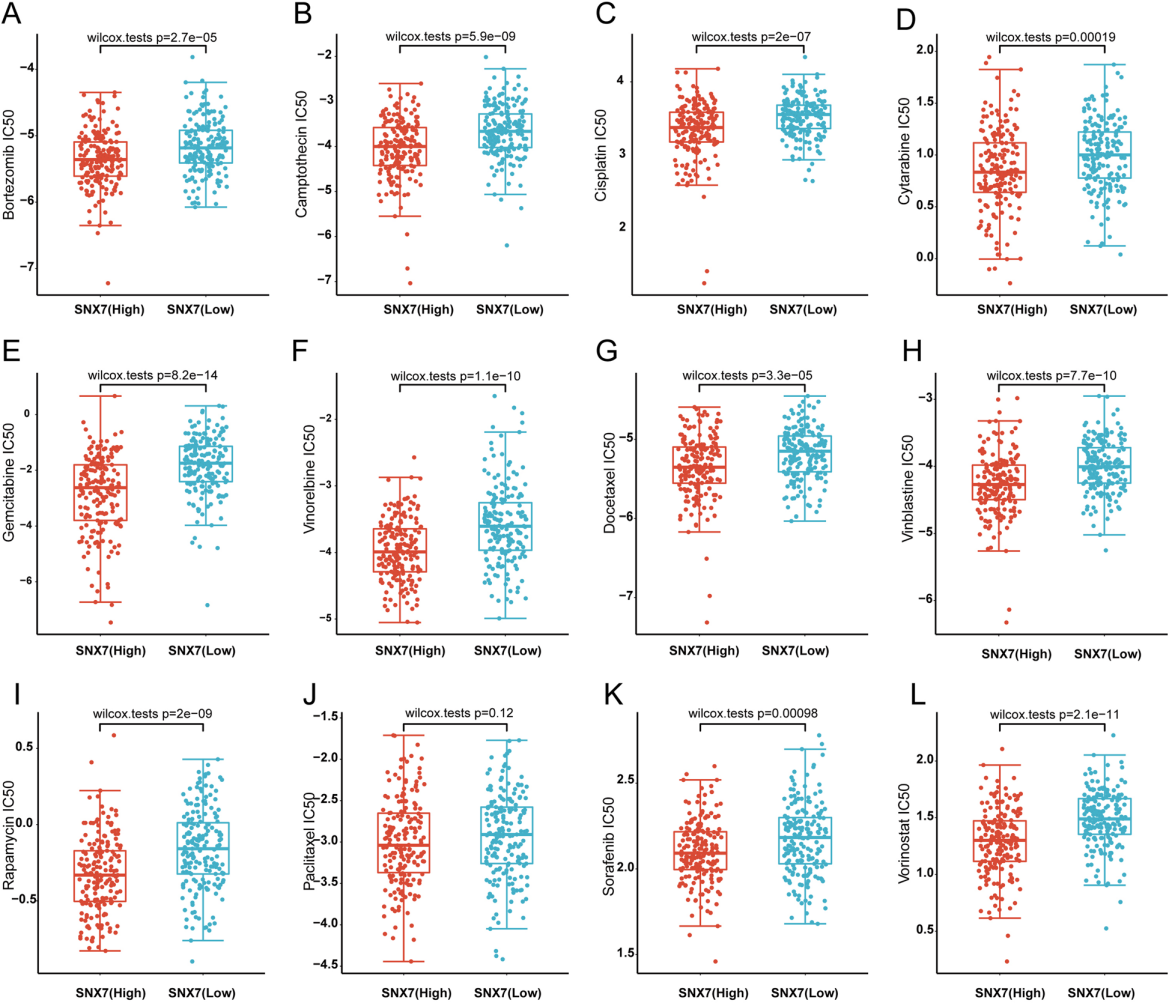
Different chemotherapeutic responses based on SNX7 expression levels in HCC. IC50 of response to chemotherapeutic drugs Bortezomib (**A**), Camptothecin (**B**), Cisplatin (**C**), Cytarabine (**D**), Gemcitabine (**E**), Vinorelbine (**F**), Docetaxel (**G**), Vinblastine (**H**), Rapamycin (**I**), Paclitaxel (**J**), Sorafenib (**K**), and Vorinostat (**L**) between the high and low SNX7 expression groups

To further understand the predictive value of SNX7 in liver cancer in response to the effect of chemotherapy, we evaluated the response of the liver cancer cells to 12 chemotherapeutic drugs by R package “pRRophetic”. Of note, the SNX7^high^ group with a lower IC50 exhibited more sensitivity to all of 12 chemotherapeutic drugs (Fig. 7A-L). To some extent, this helps to prompt physicians to provide individualized treatment for HCC patients.

### SNX7 Expression and immunology in liver cancer

**Fig. 8:**
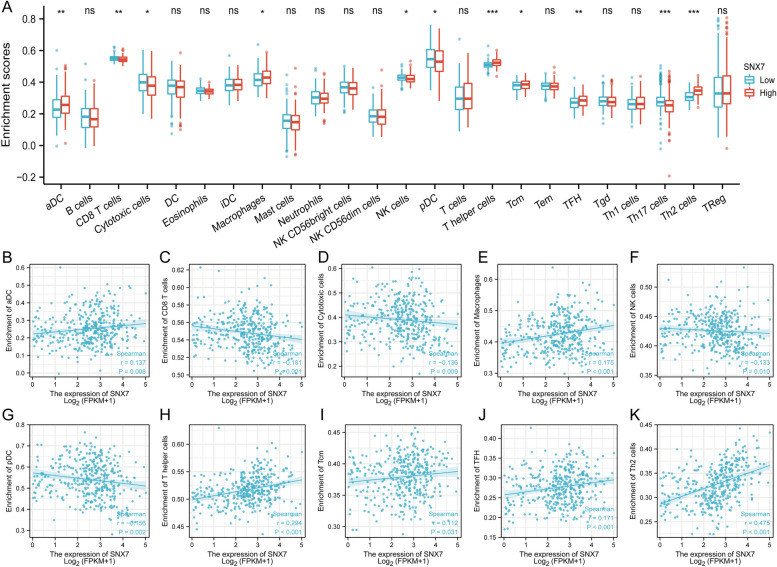
Correlation analysis between the level of SNX7 gene expression and immune cell infiltration. **A** The enrichment scores of 24 types of immune cells between SNX7^high^ and SNX7^low^ HCC groups, and Wilcoxon rank sum was applied for the significance test (^ns^ *p* > 0.05, *p < 0.05, **p < 0.01, ***p < 0.001). Correlation between SNX7 expression and (**B**) aDC, (**C**) CD8 + T cells, (**D**) Cytotoxic cells, (**E**) Macrophages, (**F**) NK cells, (**G**) p DC, (**H**) T helper cells, (**I**) Tcm cells, (**J**) TFH cells, and (**K) **Th2 cells

It has become increasingly common for cancer immunotherapy to be applied to patients in recent years [40]. Evidence suggests that immunotherapy may benefit patients with high immune infiltration in tumors, also known as “hot tumors”. Our analysis revealed that in 24 immune cell subgroups, aDC, Macrophages, T helper cells, Tcm cells, TFH cells, and Th2 cells were significantly abundant in the SNX7 high expression group, whereas CD8 + T cells, Cytotoxic cells, NK cells, pDC, and Th17 cells were remarkably reduced (Fig. 8A). Moreover, the SNX7 expression was positively correlated with aDC (*r* = 0.137, *p* = 0.008) (Fig. 8B), Macrophages (*r* = 0.175, *p* < 0.001) (Fig. 8E), T helper cells (*r* = 0.294, *p* < 0.001) (Fig. 8H), Tcm cells (*r* = 0.112, *p* = 0.031) (Fig. 8I), TFH cells (*r* = 0.171, *p* < 0.001) (Fig. 8J), Th2 cells (*r* = 0.137, *p* = 0.008) (Fig. 8K) and negatively correlated with CD8 + T cells(*r* = -0.181 *p* < 0.001) (Fig. 8C), Cytotoxic cells(r = -0.138, *p* = 0.009) (Fig. 8D), NK cells(*r* = -0.133, *p* = 0.010) (Fig. 8F), and pDC (*r* = 0.156, *p* = 0.002) (Fig. 8G).

**Fig. 9:**
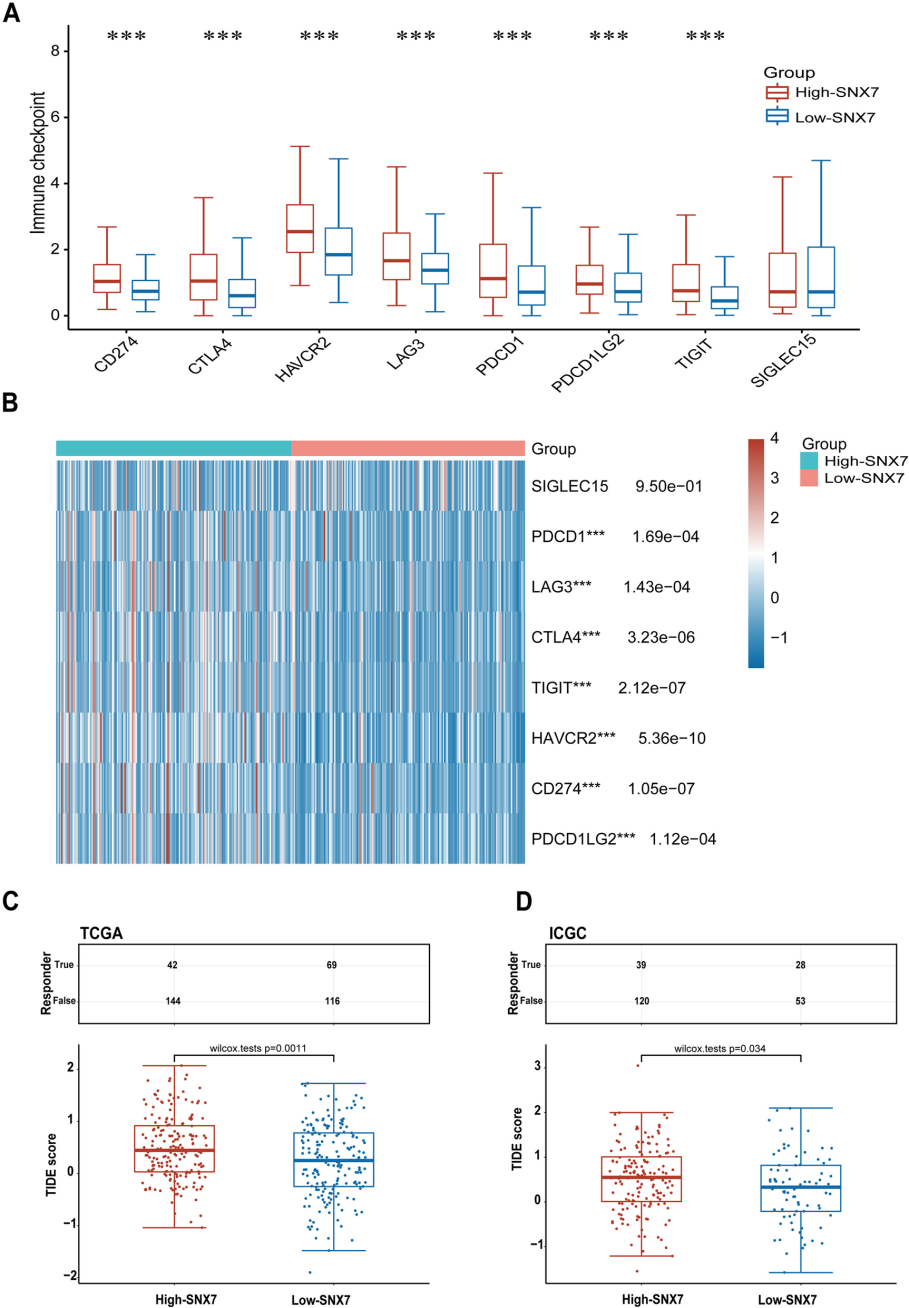
Correlation analysis between the level of SNX7 and Immune checkpoint genes or immunotherapy response. **A** Box plot comparing the expression of checkpoint genes between SNX7^high^ and SNX7^low^ HCC groups. **B** The correlation between different expression of checkpoint genes between SNX7^high^ and SNX7.^low^ HCC groups. **C**-**D** The distribution of immune response scores between high and low expression of SNX7 in TCGA cohort (**C**) and ICGC cohort (**D**). (***p < 0.001)

It is well known that immune checkpoint molecules play an important role in tumor immune escape [41]. As shown in Fig. 9A, immune checkpoint markers, such as CD274, CTLA4, HAVCR2, LAG3, PDCD1, PDCD1LG2, and TIGIT were significantly upregulated in the high SNX7 expression group (Fig. 9A, all *p* < 0.05). In addition, the heat map also exhibited that these immune checkpoint markers were positive correlation with SNX7 expression (Fig. 9B). Moreover, potential ICB response between SNX7^high^ and SNX7^low^ group were also predicted based on TCGA dataset and validated on ICGC dataset with TIDE algorithm. The data illustrated those patients with high expression of SNX7 exhibited higher TIDE scores, which indicated that the SNX7^low^ group was much more responsive to immunotherapy than the SNX7^high^ group (Fig. 9C-D, all *p* < 0.001).

### Correlation analysis of SNX7 expression and distribution of m6A related gene in HCC

**Fig. 10:**
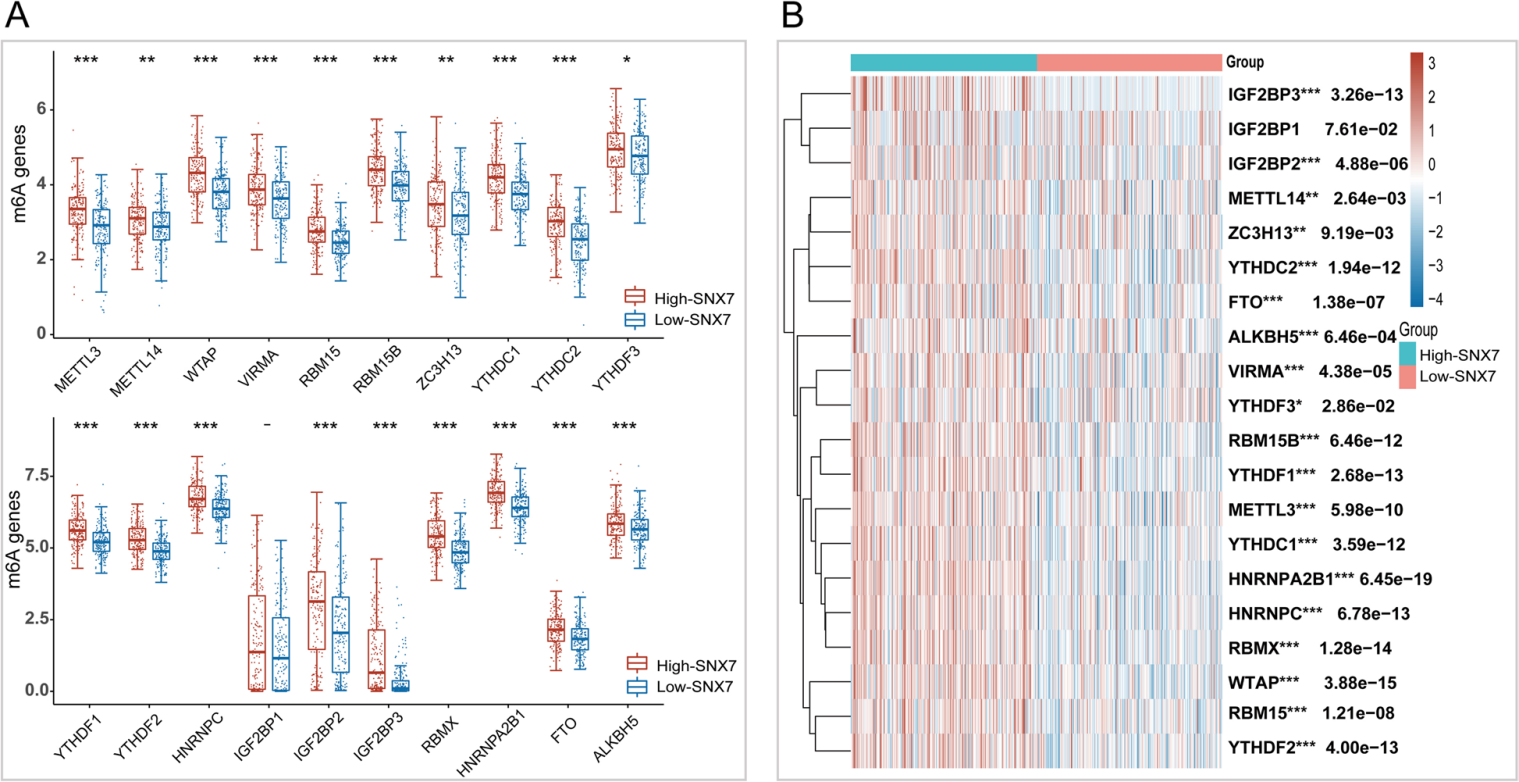
Correlations of SNX7 expression with m6A related genes in LIHC. **A** Expression of m6A-related genes in the high and low SNX7 expression groups in LIHC. **B** Heatmap shows the correlation between the expression of SNX7 and m6A-related genes. (.^−^*p* > 0.05, *p < 0.05, **p < 0.01, ***p < 0.001)

m6A modification plays a crucial role in cancer progression [42]. Based on TGGA-LIHC datasets, the correlation between the expression of SNX7 and 20 *m6A* genes was analyzed. It was observed that m6A genes except IGF2BP1 were found to be significantly higher in the SNX7^high^ group (Fig. 10A). According to the heat map of gene expression relationship, the same result as bar plot (Fig. 10B).

### Function enrichment analyses of SNX7 in LIHC

**Fig. 11:**
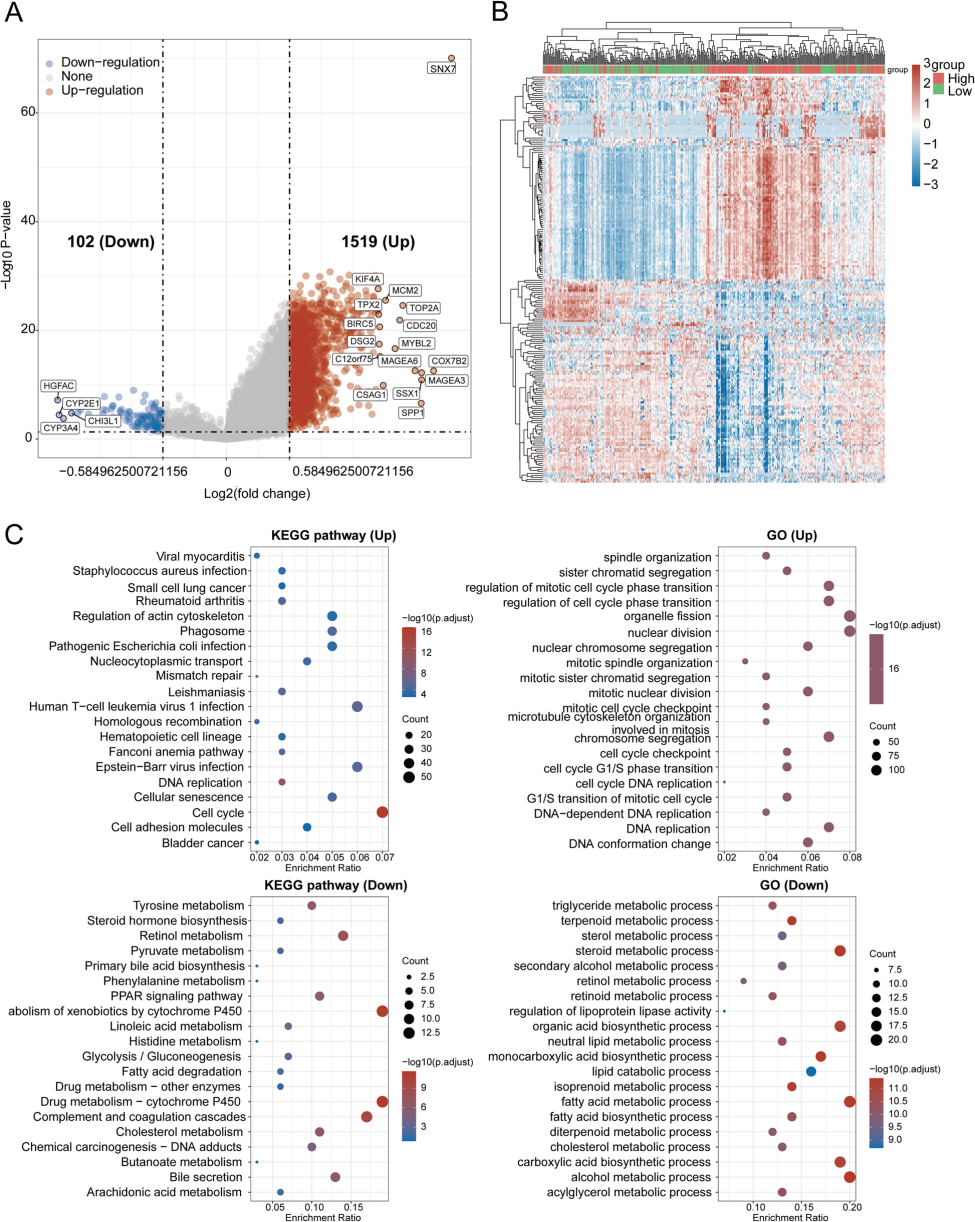
Functional enrichment analysis of SNX7 in LIHC. **A** Volcano map of DEGs. **B** The heatmap of the top 100 differentially expressed genes (50 down-regulated and 50 up-regulated). **C** The top 40 significant KEGG pathways (20 down-regulated and 20 up-regulated) and the top 40 significant GO annotations (20 down-regulated and 20 up-regulated) were enriched for SNX7-related genes in LIHC

To examine the potential function of SNX7, enrichment analyses were conducted by TCGA datasets. 1621 DEGs (FC > 1.5, *p* < 0.05) were identified that were significantly correlated with SNX7, including 1519 up-regulation genes and 102 down-regulated genes (Fig. 11A). Heatmap of DEGs were also performed (Fig. 11B). The top 20 KEGG pathways of DEGs were shown in Fig. 11C. KEGG pathway analysis identified a group of infection and immune related pathways, including Pathogenic Escherichia coli infection, Human T-cell leukemia virus 1 infection, Epstein − Barr virus infection, Staphylococcus aureus infection, and Phagosome, indicating that SNX7 biological functions are closely related to infection and immunity. Moreover, KEGG pathway analysis suggested an association with DNA replication, Mismatch repair, Cellular senescence, Cell cycle and Cell adhesion molecules. In addition, SNX7 may also be involved in tumorigenesis and development of small cell lung cancer and bladder cancer. GO analysis suggested that Cell cycle and DNA replication were identified as functional GO terms for SNX7.

## Discussion

SNX7 is an intracellular protein that directs protein trafficking decisions within the endocytic network. Although SNX7 has not been extensively studied, research has shown that it is involved in the assembly of autophagosome [43] and the processing of amyloid precursor protein (APP) [44]. It has also been found to be essential for fetal liver development in zebrafish and acts as an antiapoptotic protein in Hela or HepG2 cells [6]. Interestingly, recent studies have suggested that SNX7 may have potential value in clinical prediction of lung cancer [9, 10], which is similar to our observations in HCC. Here, we analyzed and confirmed that both the mRNA and protein levels of SNX7 are significantly higher in HCC, which is strongly correlated with the pathologic stage of the disease and can serve as an independent risk factor for HCC patients. Moreover, we found that overexpressed SNX7 levels indicated poor survival of HCC patients. Furthermore, our results reveal that the infiltration of diverse immune cells, immune checkpoints genes, and m6A-related genes are correlated with the levels of SNX7. Importantly, we discovered that HCC patients with high levels of SNX7 are more responsive to chemotherapeutic drugs such as sorafenib, but have a poorer response to immune checkpoint blockade therapy. Lastly, functional analysis indicated that SNX7 is strongly associated with Cell cycle, Cellular senescence, and Cell adhesion as well as DNA replication and Mismatch repair. Taken together, our study presents important insights into the potential role of SNX7 as a tumor biomarker in HCC.

In recent years, there has been a growing trend in using bioinformatics analyses and publicly available database to search for potential diagnostic and prognostic biomarkers in tumors. This approach was extremely popular and would be a key focus in future trials [45–48]. In the present study, we concluded that SNX7 is a potential diagnostic prognostic target for HCC by cross-validation of multiple independent datasets. we have confidence that the results of bioinformatics analysis are reliable. Considering the significant difference in SNX7 expression between HCC and normal tissues, as well as between HCC and adjacent tissues, we were curious about its differential expression between HCC and liver cirrhosis tissues. Interestingly, we observed a significant difference in the GSE25097 dataset, but not in the GSE46444 and GSE54236 datasets. Therefore, further investigation with larger, multicenter cohorts is needed to evaluate the ability of SNX7 to distinguish cirrhosis from liver cancer at an early stage. In clinical practice, there is a wide use of AFP in diagnosing, monitoring, and predicting HCC. Tumor markers like AFP are widely used to diagnose HCC. However, it is not recommended or FDA approved to monitor HCC with AFP alone [49]. Our results suggested that the SNX7 (AUC = 0.679, AUC = 0.626, and AUC = 0.626) discriminated respectively better than AFP (AUC = 0.644, AUC = 0.528, and AUC = 0.572) for HCC. In addition, we found that AFP plus SNX7 was superior to AFP or SNX7 alone in the diagnosis of HCC. Therefore, combining the two markers appears to be a promising strategy for the diagnosis and screening of potential HCC patients.

In the treatment of liver cancer, surgery combined with chemotherapy remains the primary option. However, a significant challenge in HCC therapy is the development of chemoresistance. To address this issue, identifying predictive molecules for chemotherapeutic drug sensitivity can lead to improved treatment outcomes, reduced ineffective treatments, and lowered economic burden on patients. Previous studies have shown that high expression of DTYMK in HCC is associated with poor prognosis, and HCC cell lines with elevated DTYMK expression showed increased sensitivity to multiple chemotherapy agents such as sorafenib [50]. Conversely, in colon cancer, high expression of ISYNA1 has been linked to poor prognosis and resistance to most anticancer chemotherapy drugs [51]. Our findings align with those of Guo et al.'s study [50]. However, it is important to note that there appears to be a contradiction between these results. One possible explanation for this discrepancy is that HCC patients and hepatocellular carcinoma cell lines were obtained from two separate databases (TCGA and GDSC), and the detailed chemotherapy-drug use of HCC patients in TCGA is not entirely clear. Therefore, it would be inappropriate to draw a definitive conclusion by simply combining these seemingly contradictory findings. Nevertheless, our results suggest that SNX7 may be a potential indicator of chemotherapy response in HCC patients.

The investigation of tumor microenvironments, particularly the immune microenvironment, has received significant attention in recent years. Infiltrating immune cells can reflect the immune status of tumor microenvironment and determine the efficacy of immune oncotherapy and patient outcomes [52]. In advanced liver cancer, it has been reported that the activation status of Dendritic cells (DCs) changes to immunosuppressive state [53]. In a study by Robinson et al. [54], NK cells are shown to release cytotoxic granules to destroy liver tumor cells. CD8 + T cells play an antitumor role in the tumor microenvironment, and patients with high infiltration of CD8 + T cells generally exhibit better long-term prognosis [55, 56]. Macrophages, accounting for more than half of tumor-infiltrating cells, can facilitate the escape of tumor cells from phagocytosis and immune clearance [57, 58]. It has been reported that Th1 enhances tumor immunosurveillance, while Th2 suppresses Th1 function [59]. In peripheral blood, Th17 cells are positively correlated with liver cancer progression [60]. Our results showed that activated DC (aDC), Macrophages, T helper cells, and Th2 cells were particularly enriched in the SNX7^high^ group, while the levels of CD8 + T and NK cells and Cytotoxic cells were significantly reduced. These findings suggest that SNX7 possibly has potential effect on recruiting and regulating of those immune cells within the tumor microenvironment, and thus might affect the development of liver cancer.

The high expression of immune checkpoints leads to immune evasion of tumor cells and further promotes tumor progression. The high expression of CTLA4 and LAG3 have been reported in relation to the outcome of HCC patients [61, 62]. In our study, we observed a positive correlation between the expression of SNX7 and immune checkpoint molecules. These molecules were significantly upregulated in the SNX7^high^ group. This suggests that SNX7 may play a crucial role in the regulation of immune checkpoint molecules and can potentially impact the prognosis of HCC. However, the specific underlying mechanism requires further exploration. Immunotherapy has shown significantly improvements in the prognosis of cancer patients [40], but drug resistance remains a great challenge. The TIDE algorithm has been identified as a promising tool for predicting the response to immunotherapy in various cancers [63, 64]. In our study, we found that HCC patients with low-expression SNX7 gaining a lower TIDE score exhibit a greater likelihood of responding to immune checkpoint blockade therapy. This suggests that SNX7 may have implications for predicting immunotherapy response in HCC patients.

In cancer, m6A modified genes usually play an oncogenic role [65], while m6A-related therapies, such as regulation or inhibition of m6A modifications may provide the potential therapeutic strategies for cancers [66]. We analyzed TCGA datasets and found that SNX7 expression was positively correlated with m6A modified genes. Moreover, these m6A modified genes were significantly elevated in the SNX7^high^ HCC group. These results indicated that SNX7 may potentially have a role in predicting m6A-related therapies for treatment of HCC.

In this study, we have provided a systematic and comprehensive analysis of the potential role of SNX7 in HCC, however, there are some shortcomings that need to be considered. Firstly, although we have performed cross-validated using independent datasets, there could still be some bias in the selection of these datasets. Secondly, the exact molecular mechanism underlying the involvement of SNX7 in hepatocellular carcinoma remains unclear and requires further investigation in future studies. Third, although our results suggest that SNX7 may be a potential biomarker of response to chemotherapy and immunotherapy, additional clinical validation is necessary. These considerations should be considered when interpreting the results of our study.

## Conclusion

Collectively, our findings suggest that SNX7 is abnormally elevated in HCC, associated with the immune microenvironment, and may affect the progression of HCC. SNX7 can be used as a promising novel biomarker for the diagnosis, prognosis, and prediction of response to chemotherapy and immunotherapy in HCC patients.

### Supplementary Information

**Additional file 1.**

## Data Availability

The datasets used and/or analyzed during the current study are available from the corresponding author on reasonable request.
